# Life in Suspension with Death: Biocultural Ontologies, Perceptual Cues, and Biomarkers for the Tibetan Tukdam Postmortem Meditative State

**DOI:** 10.1007/s11013-023-09844-2

**Published:** 2024-02-23

**Authors:** Tawni L. Tidwell

**Affiliations:** Center for Healthy Minds, University of Wisconsin-Madison, 625 W. Washington Ave., Madison, WI 53703 USA

**Keywords:** Ontology, Epistemology, Death, Postmortem meditation, *tukdam*, Tibetan medicine

## Abstract

This article presents two cases from a collaborative study among Tibetan monastic populations in India on the postdeath meditative state called *tukdam* (Tib., *thugs dam*). Entered by advanced Tibetan Buddhist practitioners through a variety of different practices, this state provides an ontological frame that is investigated by two distinct intellectual traditions—the Tibetan Buddhist and medical tradition on one hand and the Euroamerican biomedical and scientific tradition on the other—using their respective means of inquiry. Through the investigation, the traditions enact two paradigms of the body at the time of death alongside attendant conceptualizations of what constitutes life itself. This work examines when epistemologies of these two traditions might converge, under what ontological contexts, and through which correlated indicators of evidence. In doing so, this work explores how these two intellectual traditions might answer how the time course and characteristics of physiological changes during the postmortem period might exhibit variation across individuals. Centrally, this piece presents an epistemological inquiry delineating the types of valid evidence that constitute exceptional processes post-clinical death and their potential ontological implications.

## Introduction

‘Life in suspension with death’ is an ontological frame this work uses to signal the confluence of two distinct intellectual traditions—the Tibetan Buddhist and its attendant medical tradition on one hand, and the Euroamerican biomedical and its scientific tradition on the other. It frames how these two traditions approach the body at the time of death with different systems of not only conceptualizing life and death but evaluating what constitutes life and what constitutes death. This work looks at the epistemologies of these two traditions as they converge to answer a similar set of questions: how is mind related to body at the time of death? Can observing the dying process provide a context for the Euroamerican model in which we might investigate the potential of mind to disassociate from the brain, or for the Tibetan model the potential to observe ‘mind’ residing at the heart? And how might the time course of physiological changes—the morphochronology—of the post-clinical death period exhibit variation across individuals? This investigation focuses on an underlying epistemological discourse, namely what comprises valid evidence for each intellectual tradition generally, but specifically in determining exceptional processes at the time of death. It looks at distinctions in the recognized physical signs—classical biomarkers as employed by biomedical clinical practice nuanced for biocultural application (Worthman & Costello, 2009), compared to perceptual signs drawing from grounded theory in a specific thread of Buddhist epistemology called *pramāṇa*. As a style of South Asian discourse that propagated to Tibet starting in the 8th century CE, *pramāṇa* theory, or theory of valid evidence, was foundational for influencing the type of inquiry and diagnostics developed there by framing how direct perception (Tib., *mngon sum*) or sensory input could be relied upon to produce trustworthy knowledge of an ontological state and the related criteria for consequent inferences (*rjes dpag*) to be established (Dunne, 2004).

How each tradition defines life and death and the respective systems of evidentiary signs upon which they rely integrally influence perceptions of the transition from life to death and the related implications of each state. They also delineate how that transition might be characterized as a gradual dissipation of life at death, a pronounced definitional moment changing life to death, or perhaps a diffuse ontological liminality of life “suspended” in death—that is, the relative “boundedness” of each state. As such, this paper centrally explores two distinct biocultural ontologies—a post-death meditative equipoise state on one hand, and effervescent-like attenuated postmortem decomposition on the other.

First, this article will describe the historical background on the Tukdam Study as well as a brief introduction to the ontological and epistemological lenses of the Tibetan and Euroamerican traditions of inquiry. Next, it will present an ethnographic account of the longest *tukdam* state examined by the research team to date. This case will form the basis upon which we will discuss particular biomarkers and perceptual cues assessed by each tradition in the collaborative investigation. Subsequently, we will examine a second case study abbreviated in duration, but with attendant signs seemingly ordinary to the Euroamerican tradition yet extraordinary to the Tibetan tradition. Finally, we will reflect on the attendant ontologies and epistemologies that frame differential perspectives on life and death for these two intellectual traditions.

## Background

The Tukdam Study arose out of a conversation between affective neuroscientist Richard Davidson and His Holiness Tenzin Gyatso, the XIV Dalai Lama in 1995 during one of the numerous exchanges between scientists and Buddhist scholars held in Dharamsala, India. The Dalai Lama spoke about a meditative state achieved at the time of death, called *tukdam* (*thugs dam*), in which the practitioner seeks to achieve ultimate realization into the fundamental nature of mind. In this practice, he described, the practitioner—usually residing in an upright meditation posture postmortem—maintains a suppleness, elasticity, and radiance to the skin and complexion; elicits a persistent warmth in the body most pronounced at the heart region; emits a fragrant odor; and suspends the normal chronology of physiologic processes at the time of death. Ontologically, Tibetan Buddhists recognize that, in this state, the coarse and subtle levels of body and mind have dissipated and a very subtle level of mind along with a very subtle level of ‘body’—energetic activity, known as ‘winds’ (*rlung*)—are present. The Dalai Lama explains that when the practitioner concludes this type of meditation, voluntarily releasing the state, the body will begin to effuse a putrid odor and signs of decomposition will begin to appear. He describes this state like “someone asleep” yet “no longer breathing, like a corpse but, unlike a corpse, not smelling” (Dalai Lama XIV, 2006, p. 201).

The XIV Dalai Lama has championed the preservation of Tibetan cultural heritage and the endowments it offers the global community in greater mental health, well-being and sustained happiness through rich contributions to contemplative thought and practice (Davidson, 2021; Dunne, 2015; Lutz et al., 2007). In this effort, he has actively supported and engaged in collaborations with scientists to investigate physiologic and psychologic changes that these contemplative practices engender to provide evidence of their benefits and encourage greater integration of practices supporting happiness and well-being globally. He has framed such initiatives under a paradigm of universal responsibility to support greater global wellness, and a commitment to uncovering mutually agreed upon verifications of such determinants through rich conversations across different intellectual traditions, epistemological angles, geographic populations, religious paradigms, and cultural frameworks. This study is motivated by many insights and achievements produced by contemplative neuroscience research over the last several decades (Davidson, 2021) and particularly those specific to studies of long-term meditators (Brefczynski-Lewis et al., 2007; Chaix et al., 2020; Ferrarelli et al., 2013; Fucci et al., 2018; Kaliman et al., 2014; Khalsa et al., 2008; Lutz et al., 2008, 2009, 2013; Rosenkranz et al., 2016; Wielgosz et al., 2016). However, even more so, this study is animated by the XIV Dalai Lama urging members of each intellectual tradition to engage in an intrepid collaboration that forges epistemological bridges and cultivates an integrated inquiry into the nature of mind, the nature of body, the nature of living, and the processes, experiences and potentialities of dying.^1^

Yet what can *tukdam* tell us about well-being through the life course or conceptualizing an ideal death cross-culturally? For the Tibetan context, *tukdam* epitomizes a lifetime of advanced practice investigating the fundamental nature of mind that, utilizing a key temporal and ontological window—the moment of death—capitalizes on a specific psychophysiologic context in which the coarse body and subtle mind disassociate—to facilitate the greatest possibility for apprehending this reality and thus, actualize spiritual realization. The ultimate aims of such practices are to cultivate a capacity of wisdom and compassion such that all activities benefit other beings. Likewise, those who have achieved such realization represent the ultimate cultural exemplar of immutable well-being. Layperson cases of *tukdam*, both men and women, range from those with extensive formal meditation training to those who exhibit particular qualities of devotion and compassion, committed views of benefiting others, and aim to gain realization in future lives to benefit others even more extensively. From the Tibetan perspective, investigating *tukdam* might provide scientists an opportunity to witness the unique qualities of mind of such a practitioner and a context in which potential evidence might be ascertained that consciousness need not be generated from the brain.

For the Euroamerican frame of reference, and for the Western scientific tradition, this state provides an opportunity to observe the possible persistence of brain activity following cardiac and pulmonary cessation as observed in a small sample of intensive care unit patients taken off life support and in various animal models (Auyong et al., 2010; Borjigin et al., 2013; Grigg et al., 1987; Norton et al., 2017; van Rijn et al., 2011; Xu et al., 2023), as well as potential systemic activity such as in the hypothalamo-pituitary axis (Arita et al., 1993). For forensic science, it also provides the possibility to witness variation in the normal chronometry of the postmortem process, explore variation in odors emitted (Dekeirsschieter et al., 2012; Vass, 2019; Verheggen et al., 2017), examine other potential markers of suspended animation such as dampening of insect and microbial activity (Javan et al., 2016, 2019; Ventura Spagnolo et al., 2019; Wells, 2019), and posit the physiological processes facilitating such markers (e.g., Fryer & Brown, 2015). It also offers the opportunity to reflect on potential subjective experiences that persist after clinical death (e.g., Vicente et al., 2022) that are elaborately detailed in classical texts of the Buddhist tradition.^2^

The Tukdam Study, formally began in 2007 as a collaboration with the Office of His Holiness the Dalai Lama, Men-Tsee-Khang—the Tibetan Medical and Astro-Science Institute under the auspices of the Dalai Lama, its biomedical partner Delek Hospital, and the University of Wisconsin-Madison. The full protocol currently applied was confirmed in 2012 as ‘FMed’, or the ‘Field Study of the Physiology of Meditation Practitioners and the Tukdam Meditative State’, whereby the study investigates healthy baselines of senior practitioners in Tibetan Buddhist monasteries throughout India, tracking them in their elderly years with the hopes of following them through their transition at the time of death. It also examines postmortem cases as soon as the team is notified of their occurrence. From inception, the aim of the study has been an empirical long-term research project that would integrate psychophysiological and ethnographic research to examine the physiological correlates of this state and explore the practices that Tibetan Buddhists describe as facilitating the potential for entering into it. The collaboration has also sought to investigate the biological and neural mechanisms that might be involved in the physical signs observed.

Though no recognizable electroencephalographic activity has been discernable in any *tukdam* cases recorded to date, the earliest case assessed was already 26 h postmortem (Lott et al., 2021), beyond the temporal window predicted to witness such neurophysiological functioning, even in terms of brainstem response (Facco et al., 2002; Garcia-Larrea et al., 1987) and potential auditory pathways responsive to stimuli moments prior to clinical death (Blundon et al., 2020). Ultimately, the Tukdam Study has aimed to observe practitioners as they transition in the dying process to assess the initial period through clinical death. However, cultural definitions of the *tukdam* state often require leaving a body undisturbed for 3 days postmortem before definitive confirmations of a *tukdam* state are declared, since even non-practitioners might have remaining subtle consciousness in the body and should be left for 84 h or 3.5 days before cremation (Desi Sangyé Gyatso, 1996 [17th century CE]; Samdhong Rinpoche, 2017).

In November 2020, the Office of His Holiness requested the Tukdam Study form a larger collaboration with neuroscientists from the Russian Academy of Sciences and Moscow State University. The Russian team had expressed interest in investigating *tukdam* and its related meditation practices and had initiated bringing monastics to Russia from Tibetan monasteries in South India, namely, Drepung, Sera, Gaden, Tashi Lhunpo, and the Higher and Lower Tantric Colleges of Gyutö and Gyudmed, to train in brain imaging techniques relevant to the study. Most notable among these recruited monastics were the Tenzin Gyatso scholars who trained in physics, biology, and history of science at Emory University as part of the Emory-Tibet Science Initiative founded by Geshe Lobsang Tenzin Negi under the sponsorship and direction of the XIV Dalai Lama from 2009 to the present.

In 2019, the group of talented science-educated monastics that the Russian team recruited provided the Tukdam Study with a new generation of research partners in addition to the Tibetan medical physician teams contributed by Men-Tsee-Khang who served as the primary field team previously. This collaboration facilitated a context for these cultural experts in the medical system that codeveloped with Tibetan Buddhism for over a millennium—Tibetan medicine—to work alongside colleagues rigorously trained in dialectical theory of the contemplative system and the related meditative states. These two arms of cultural experts provide knowledge on both the clinical and contemplative applications of an integrated epistemological and ontological system to the study. Monastic field team members now serve as the primary field investigators applying the psychophysiologic and neuroscientific instrumentation on *tukdam* cases, negotiating the cultural sensitivity and contextual interstices within their own community, and Tibetan physicians now lead only the health-related data procurement minimizing interruptions to daily clinical duties when a case arises. The US-based and Russian research team leaders provide advisory roles and are largely absent as a field presence during cases. Forensic specialists from both Russia and the US were recruited to employ a battery of postmortem forensic measures, including several for skin tone and tissue integrity developed for the study itself. With this expansion, the Tukdam Study team now numbers over 60 members, more than half of which are the Tibetan monastic and physician field researchers.

## Tibetan Buddhist Understandings and Ontologies of *Tukdam*

*Tukdam* has been widely recognized historically in the Tibetan cultural world as one of the unexcelled final meditation states for an advanced practitioner’s life achievements.^3^ Yet its explicit mention in the textual corpora of the Buddhist canon—the Kangyur, or scriptures of the Buddha’s word; the Tengyur, or commentaries on those scriptures; the Abhidharma, or the compilation of Buddhist psychology, philosophy, and metaphysics; the Revealed Treasures, or texts hidden to be revealed for applicability in later times; and later commentaries as a psychophysiological state with specific signs is relatively minimal (Ngawang Jinpa, 2020, v, 489–490).^4^

Though many scriptures in the Buddhist canon detail the internal phenomenological signs a practitioner can expect in the transition to death, the account best known to the English-speaking world is the 1927 translation of the *Great Liberation Upon Hearing in the Intermediate States*^5^ compiled by 17th century treasure-revealer^6^ Rigdzin Nyima Dragpa (1647–1710) from Karma Lingpa’s (1326–1386) 14th century revealed treasure of *The Peaceful and Wrathful Deities: A Profound Sacred Teaching on Natural Liberation through Recognition of Enlightened Intention.*^7^ In English, it is known simply as the “Tibetan Book of the Dead” (Evans-Wentz, 1927).^8^ This text provides a detailed first-person account of the phenomenological, physical, and psychological signs of the dying process and the transference of consciousness through several intermediate states before reincarnating into the next lifetime.^9^ It also provides explicit instructions for the key points in the process that offer contextual opportunities for ultimate realization or enlightenment, such as in the case of *tukdam*. *The Peaceful and Wrathful Deities* stems from the system of the Guhyagarbha Tantra, or Tantra of the Secret Essence, a principal textual cycle studied by the Nyingma School of Tibetan Buddhism, which is the oldest school of Buddhism in Tibet, tracing its lineage to the first wave of Buddhist teachings transmitted to Tibet from India. This cycle is one of the most revered systems instructing practices for achieving a realized state for this school (Mipham, 2009). One of the earliest historical figures documented as exhibiting characteristics of *tukdam* is that of Longchen Rabjam Drimé Özer (1308–1364)^10^ (Nyoshul Khen, 2005, p. 131), who also composed an important commentary series to the Guhyagarbha Tantra^11^ and was known as one of the greatest Dzogchen masters in the Nyingma tradition (ibid: 98).^12^

However, the current study has largely focused on Geluk institutions and practitioners due to the Dalai Lama’s patronage as one of the highest lamas in the Geluk school. The practices of the Geluk school that facilitate entry into *tukdam* are distinct from those of the Nyingma. The second earliest historical figure documented as residing in *tukdam* is that of the founder of the Geluk school Je Tsongkhapa, as written by one of his closest disciples Khedrup Je ([15th cent. CE], 2021) and for whom the Guhyasamāja cycle of practice is considered central. The other primary practices of senior practitioners in the Geluk school and those mentioned as central for those who entered the *tukdam* state are the tantric cycles of Cakrasaṃvara and Vajrabhairava. However, Guhyasamāja tends to be the dominant practice among most *tukdam* cases documented to date and that which Tsongkhapa was revered for propagating.

The Guhyasamāja, also known as the King of Tantras, belongs to the Yoga-niruttara or Highest Yoga Tantra class of Tibetan Buddhist practice. This class of practice involves an initial stage in which the practitioner visualizes a dissolution of their mundane form, rearising in the form of a deity or a multiplex of deities; and a final stage in which a subtle anatomy of that form is visualized involving manipulation of channels, energetic centers and seminal essences to engage certain qualities of mind. In the concluding phase, the practitioner dissolves the entire visualization into emptiness.^13^ The degree of and stability in visualization and nuanced manipulation of aspects of the form are considered central to the production of a prolonged *tukdam* state (Dalai Lama XIV, 2003, p. 150) for Geluk practitioners. The Guhyasamāja practice cycle specifically includes visualization of a maṇḍala of 32 deities,^14^ which, as with all Buddhist tantric systems for which Tibetan Buddhism and its attendant ontology are derived, provides a visual representation of the components of the enlightened mind through distributed form.

The maṇḍala demonstrates a key ontological difference between the Tibetan Buddhist perspective of the body-mind complex and that of Euroamerican biomedicine that locates mind as an epiphenomenon of the brain (Cobb, 2020). A maṇḍala details the symbology, or blueprint map, of an individual’s psychophysiological constituents comprising purified forms and qualities of interrelated aggregates (*phung po*). These aspects constitute both the person and the enlightened qualities thereof. The maṇḍala is the most elaborate and technical characterization of the self. It provides high resolution detail to an idealized self, which, in its mundane form is the simplified framework of self as five psychophysical aggregates: the aggregates of form, feeling, discernment, conditioning factors, and consciousness (Ju Mipham [19th cent.], 2004, p. 16; Dalai Lama XIV, 2020a, p. 493). Simply put, the aggregates can be thought of as the body, physical world, five senses and processes of cognitive activity; motivational tendencies; conceptual labeling and discrimination between objects; feelings; and an underlying awareness or consciousness (Dalai Lama XIV, 2022, p. xiii). This is the collection of entities that comprises the person from the Buddhist and Tibetan medical ontology.

Although other schools of thought in South Asia during the time of the Buddha posited the existence of an independent essence or soul separate from body and mind that comprised the person, all Buddhist schools refute the existence of an unconditioned, autonomous entity as such. The Buddhist perspective of a self is an interdependent dynamic relationship between physical and mental attributes of an individual—the psychophysical components just described—to constitute a person as embedded in a vast network of socioecological relations. The cluster of dependently arising mental and physical events often interpreted or ‘appropriated’ as a ‘self’ is due to apparent continuity. However, the experience of their continuity itself, is also considered dependent.

Whether in the Euroamerican scientific or Tibetan intellectual tradition, the continuity of consciousness after death integrally relates to the concept of what constitutes a person—the ‘self’ or ‘I’. The five aggregates provide one such mundane representation of the Buddhist ontological self, and the maṇḍala provides the nuanced details of that representation in supramundane form. In this paradigm inhere two interdependent, causally engaged ‘self’ concepts: one that comprises a gross body and conditioned mind, and one that is a subtle body, or minimally perceptible physical energy, and subtle mind, or consciousness, indistinguishable as two facets of one phenomena (Dalai Lama, 2020a, p. 288, 2022, p. xiv; Dunne, 2020, pp. 202–203). The subtle mind has the quality of awareness, and the subtle body is the force, or dynamic substrate, that enables, mobilizes, and activates the mind, described as subtle wind. In this cycle of practice, these two are inextricably conjoined and are described as the ultimate nature of a person or the fundamental nature of mind. This Buddhist ontological view shapes why it is understood that a practitioner’s engagement of the most subtle aspects of mind in the dying process is seen to affect the most subtle activities of the physical body that alter even the physical processes of decomposition and allow for a minimal level of activity at the heart as residual support.

## Euroamerican Biomedical Understandings and Ontologies of Death as Event or Process

Though the heart as the principal organ governing human life and managing vital activities was accepted for centuries in Europe (Le Goff, 1989), during the eighteenth and nineteenth centuries, investigations in vivisection and physiological experiments led to a decentralized view of that which managed vitality, somewhat similar to the distributed view of mind described above in the Tibetan Buddhist and medical perspective. For example, early eighteenth century French naturalist Georges Buffon proposed that all creatures comprised living “molecules” and that these separate entities summate into the organization that animates life (Lock, 2001, p. 73). The decentralized view contradicted that which was promulgated in the 17th century by René Descartes who suggested all control was concentrated in the single organ of the pineal gland (Pernick, 1988, p. 28). Even in the late eighteenth century, physiologist Marie François Xavier Bichat proposed that the body comprises “vital tissues” and that “organic life” resides in the heart, lungs, kidneys, and other organs, and “animal life” in the brain, which he suggested also produces sensation and volition. As Margaret Lock describes in her analysis of how organ transplant practices reshaped conceptualizations of death, these historical arguments continue to animate a discussion on the “location” of a person, and “when and under what conditions person and body cease to exist” (2001, p. 73).

Whether death is understood as an “event” or a “process” in the Euroamerican tradition also informs perspectives of life as produced through centralized or decentralized mechanisms (Gervais, 1986; Laureys, 2005).^15^ Regardless of the lack of global consensus (Wijdicks, 2002), the current practical clinically deployed definition of death in the biomedical clinical and forensic settings has focused on brain stem death (Laureys, 2005, p. 900). This practice has largely been implemented, as Lock has shown, for facilitating decisions around the removal of life support and retaining viable organs for donation (2001). As such, death has become an ‘event’ not a process. This approach has negated time course progressions many observe for the dying process and has effectively removed concepts of ‘mind’ from the purview of biomedicine, relegating it to metaphysics and philosophy (Cobb, 2020).

Segregating the mind concept from biomedical contexts presents one of the challenges of two ontologies lacking even basic mutually informative conceptual frameworks with which to engage since the concept of mind critically informs the entire ontological paradigm for *tukdam* as a phenomenological state in the Tibetan tradition. Likewise, the lack of investigative means in affective and contemplative neuroscience to understand cognitive-affective processes that are only accessible by first-person accounts led many neuroscientists in the field to seek first-hand contemplative experience to understand such epistemic methods in gaining insights into ‘mind’—specifically related to core concerns of cognition, perception, and behavior (Hasenkamp, 2014). Thus, neurophenomenology emerged to investigate mind, consciousness, and experience through various lenses, including first-person introspective, that provide differing understandings on the embodied nature of mind (Varela, 1996).

## Signs of Physiological Changes: Biomarker and Perceptual Cues

Both Tibetan medical and Euroamerican biomedical systems rely on indicators from each tradition’s respective ontological paradigms and epistemic diagnostic instruments to track pathways linking a physiological outcome to the factors that influence it. For the Euroamerican tradition, these indicators are measurable physical features called biomarkers used to detect effects of context or underlying physiological processes (Worthman & Costello, 2009, p. 284). In the biomedical context, a biomarker is considered an objective measure evaluated as “an indicator of normal biological processes, pathogenic processes, or pharmacological responses to a therapeutic intervention” (Biomarkers Definitions Working Group, 2001, p. 91). An example is blood glucose for diagnosing diabetes mellitus. The validity of a biomarker is how accurately it predicts the impact of an event or intervention on the outcome. However, as Worthman and Costello describe in their seminal work on biocultural approaches to biomarkers, biomarkers are necessarily imperfect due to the multiple pathways that mediate and modulate any health outcome (2009, p. 286). As such, biomarkers represent an estimation of actual physical states and physiological processes, not a precise signifier.

For those researchers who attend to the biocultural and contextual determinants of health, biomarkers that draw upon cumulative, broad-spectrum effects on physiological outcomes are ideal. An example is psychosocial stress which draws on a variety of mental and physical health sequelae, including altered regulation of the hypothalamo-pituitary-adrenal axis, immune system and metabolic pathways (Worthman & Costello, 2009, p. 286). The construct of allostatic load was developed from an index of measures related to contributory functions and systems affected by stress and indicate an aggregate resultant outcome of impaired health (Panter-Brick & Worthman, 2008).^16^

With a similar interest in tracking the processes that mediate a context, event, or intervention and its outcome, Tibetan medical physicians and Buddhist adepts have relied on perceptual and inferential markers derived from the *pramāṇa* tradition as a discourse assessing the validity of evidence largely gained through perceptual and inferential means. Such an inheritance of intellectual history allows them to characterize consciousness—phenomenologically from the practitioner’s side, but also empirically from signs apprehended by an external observer. Percepts classified according to discrete descriptive metaphors are perceived and interpreted through guided mentored experience. Since perception is considered a causal process in this system, the individual learns to cultivate direct perception as a nonconceptual cognition vis-à-vis a delineated aim for achieving a specified goal. For example, for the purpose of reestablishing wellness, an individual would only access perceptual content relevant to identifying the underlying causes that are inhibiting a state of wellness, such as a particular disease.^17^ In doing so, one might investigate the conditional influence of psychosocial stress on disease progression by assessing characteristics in the pulse classified as “hollow,” “buoyant” and “intermittently stochastic” in its flow. Such characteristics relate to mental disturbances Tibetan diagnostic theory recognizes as having specific measurable physiological repercussions. The externally observed changes are connected to internal phenomenological states of feeling untethered from the ground, buoyancy in one’s body, diminished concentrative capacity, impulses for constant movement and an urge to seek sensory fulfillment externally. This system of tracking physiological, psychological and experiential changes relies on perceptual cues, some of which are accessible to a third person observer, and some of which only available to the subjective experience.

The epistemological lenses of these two traditions determine what kinds of signs each recognizes for specific processes and progressions, say during the dying process, and the ways in which each conceptualizes and then confirms the final transition to death. Euroamerican biomedical and research communities increasingly rely on biomarkers that index function and dysfunction that, at the time of death, focus on brain stem, heart and lung parameters to demarcate end of life. Subsequent physical changes are dismissed after this “biological” barrier is reached from which the domain of metaphysics is assumed to take over. The Buddhist and Tibetan medical approaches track the release of the consciousness from the dying body through perceptual markers that integrate psychological and physical indicators. These epistemic systems guide the recognition of physiological changes as mundane or supramundane and posit the effects of the conscious mind on physical form—even for teleological interpretation as indicating enlightenment, realization, luminosity.

Although the Euroamerican tradition does not recognize biographical details of an individual as relevant to the decompositional changes observed in the postmortem period beyond recent medical history and dietary and medication intake, those recognizing *tukdam* in the Tibetan tradition often place great significance on the biographical details. Hence, in the ethnographic examples presented, a rich characterization of the individual can frame the signs observed in the postmortem period. This will also become relevant to how the two intellectual traditions negotiate the significance of postmortem signs within each respective epistemology.

## Tracking Signs of ‘Normal’ Postmortem Chronology

In the normal postmortem chronology from the Euroamerican biomedical paradigm, a paling of the skin generally occurs within the first 15–25 min after clinical death, a stage called pallor mortis or the “paleness of death.” Though the paling is most pronounced in those with light or white skin (Schäfer, 2000), the collapse of capillary circulation throughout the body induces a loss of vibrancy across skin tones. During the first 30 min to 2 h, regions on dependent surfaces manifesting blood pooling, known as livor mortis, become evident through the change of tissue color, which reaches its maximum coloration within 8–12 h after clinical death (DiMaio & Molina, 2021, p. 17). Warmer temperatures can accelerate the process, and cooler temperatures can delay it up to 36 h.

Within the first 2–12 h the body stiffens in rigor mortis to its peak as myofibrils of the muscle cells—after glycogen stores are exhausted—deplete in adenosine triphosphate (ATP), the molecule that provides energy for muscle contraction, neural signaling, and other forms of biochemical energy transfer. ATP depletion instills rigidity in the body, but when the myofilaments start to decompose, the body relaxes again, a process that generally unfolds after 24–48 h with an average of 36 h in temperate climates (DiMaio & Molina, 2021, pp. 19–20). The disappearance of rigor mortis tends to accelerate with greater rates of decomposition when the bacterial flora of the gastrointestinal tract spread throughout the body and produce putrefaction.

The common sequence of events in decomposition starts in the first 24–36 h with greenish discoloration in the abdominal lower quadrants, the right liver side more pronounced than left (DiMaio & Molina, 2021, p. 23). The greenish color tends to progress to neck, shoulders, and head with swelling of the face, eyes bulging and tongue protruding due to bacterial gas formation and marbling. Marbling refers to vasculature breakdown as hemoglobin and hydrogen sulfide react producing greenish-black coloration along blood vessels.

Generalized bloating tends to occur within 60–72 h, followed by vesicle formation, skin slippage and hair loosening. At this point the body is usually pale green to green-black in color. The face also begins with a pale greenish color and then changes to greenish black, then eventually distinctly black. Purge or decompositional fluid normally drains from the mouth and nose thereafter. Cold weather slows or even stops decomposition; however, the above chronomorphology is expected for bodies in most temperate climates.

Flies tend to be the most common insect associated with decomposing bodies, depositing eggs in body orifices and open wounds immediately after death. A general rule of thumb is a body found with only eggs present, is 1–2 days since death. It can take 6–10 days for maggots to grow to pupa stage after hatching; adults tend to emerge in 12–18 days. Variation depends on geographic location and meteorological conditions (Byrd, 2009, p. 135).^18^

Visual observations and body manipulations assessing pallor, livor, and rigor mortis states along with rule-of-thumb assumptions correlating to expected duration of each tend to be the most common biomarkers measuring postmortem changes and estimating time since death (DiMaio & Molina, 2021, pp. 20–21). However, forensic pathologists have historically used other measurements as well. When the sequence of insects colonizing a decedent is known for a given area and set of circumstances, an analysis of arthropod fauna can provide an accurate and precise method for estimating elapsed time since death (Byrd, 2009, p. 201). When such an analysis is not possible, other evaluative methods include:

- Rectal temperature;^19^
- Gastric emptying estimates;^20^
- Potassium quantitation from vitreous fluid between eye retina and lens;
- DNA, RNA, and protein degradation in body fluids and tissues;^21^
- Microbial flora changes;^22^ and
- Volatile organic compound (VOC) emission composition and rate.^23^

However, the above measures are highly sensitive to ambient temperature and humidity and cannot be relied upon for accurate assessments of time elapsed since death.^24^

In each case the Tukdam Study has assessed, the team forensic experts have kept the biomarkers for this ‘normal’ postmortem chronology in mind and how particular signs signal each phase. However, many of the mainstream forensic methods cannot be implemented in the particular cultural context in which the study is conducted. Due to the cultural importance of using non-invasive methods, the study relies on unconventional approaches to particularly characterize the progression (or lack thereof) of pallor and livor mortis vis-à-vis visual metrics. To measure color changes related to pallor and livor mortis and develop a way to quantify the Tibetan quality of “radiance” (*mdangs*), the team forensic anthropologist implemented a colorimetric approach drawing from the Munsell color system.^25^ The system, developed in the early 20th century as a standardized color system for soil research, has been applied in forensic contexts for descriptions of skin, hair and eye color in forensic pathology (Reeder et al., 2014). The system draws on three independent properties of color—hue, chroma, and value—from which Munsell produced scales of samples that change at a perceptually uniform rate to the observer (Cochrane, 2014). The scales were deployed in the field along with a white balance color calibration tool used in videography to standardize color assessments across cameras.^26^

## The 37-Day Case

In February 2021, the onset of the SARS-CoV-2 pandemic necessitated that the Tukdam Study team shift to a highly streamlined image-focused protocol to navigate restrictions across India of lockdown, quarantines in the monasteries, limited case access, and cessation of all international travel despite local field members. Just 1 month later, despite the highly constrained conditions, the study had its longest case in the project history—a senior Buddhist practitioner remained in the post-death meditative state for 37 days, during which his body demonstrated marked resistance to the decomposition process; his skin retained elasticity, pliancy, and tone; and his complexion presented with warmth in hue and only minimal discoloration at hands and feet.

The monk was born in 1935 in Markham, a small village in southern Kham located along the southeastern most region of the Tibetan Plateau, and was recognized as a reincarnation of Maitreya, the future Buddha, particularly by those in his home region. At the age of 11, he received his novice monk ordination at Palden Lhura Monastery, founded by a Je Tsongkhapa disciple. At age 19, he entered advanced studies in Lhasa at Drepung Loseling Monastery, one of the most renowned monastic universities in Tibet. At age 21, he received full ordination vows from the XIV Dalai Lama’s tutor Ling Rinpoche, who passed in the *tukdam* state in 1983. After completing his advanced examinations conferring the highest degree recognition of *geshe lharampa*, he entered the upper tantric college of Gyutö Monastery. During this time, he underwent rigorous training in ritual performance studies, sand maṇḍala construction, elaborate butter sculpture and *torma* cake offerings, stupa development and detailed iconometry for all related ritual practice arts. The blueprints for these ritual objects are considered a critical support for practice and potent in themselves, since proper construction according to the exact measures is thought to not only confer spiritual transformative potency to the viewer, but imbue capacity for more vivid and precise visualization in the producer (Ju Mipham [19th cent. CE], 1975).^27^

Guided by senior and junior tutors to the XIV Dalai Lama, he received the oral transmissions of the commentaries and teachings related to the Guhyasamāja Tantra cycle for which the maṇḍala is one of the most elaborate among tantric cycles. In addition to Guhyasamāja, he also studied detailed and concise measurements for the maṇḍala ritual supports for all four classes of tantra in the Geluk School—Kriyā, Caryā, Yoga and Yoga-niruttara.

In 1959 with the invasion of Tibet, he followed the XIV Dalai Lama into exile, eventually settling in Bomdila where Gyutö Monastery was established in exile. There he became the monastery ritual specialist preserving and reviving the construction and drawing of mandalas, the related tantric practices, and the ritual offerings particularly for the three great central deities, or *yidam*, in the Geluk tradition—Guhyasamāja, Vajrabhairava, and Cakrasaṃvara, as well as the dharma protector Mahakala.^28^

The monk developed heavily illustrated curricular resources critical in laying the foundations for tantric practice and its rituals not only for monastic students but all practitioners. Monastic enrollment in the monastery increased and the monastery became recognized for exemplary monastic disciplinary conduct, artisanry skills and ritual performance. Reincarnate lamas and the highest learned scholars traveled from afar to learn the specialty curriculum as he became a widely recognized teacher.

Although he had already engaged in extensive practice in the generation stages, or self-visualizations, of the Geluk School’s three principal tantric cycles, in 2004, just before turning 70, he entered 3-year retreat on the 13-deity form of Vajrabhairava, considered a destroyer of death. After retreat, he continued working on the tantric ritual curricular resources, but spent increasing amounts of time in semi-retreat, due to a deterioration in his condition from years of diabetes and hypertension. During this time, he focused primarily on Guhyasamāja.

On January 14, 2021, at the age of 86, the monastery awarded him its highest teaching honor for lifetime contributions to tantric education. Yet soon after, he fell sick with COVID-19. Instead of remaining at the hospital, he requested to return to the monastery. In a cement room quarantined from the rest of monastery with a single bed, attended only by a solitary attendant to minimize exposure, he rested and passed. When he took his last breath and the attendant felt his pulses cease, it was 4:40 pm, on the 27th day of the 1st month in the Iron Ox year of the Tibetan calendar, March 10, 2021 of the Gregorian calendar. He was reclined when he passed, not the classic upright meditative posture or full lotus. However, the signs were still considered remarkable by the Tibetan Buddhist and medical experts.

His disciples described that the act of dying and the subsequent signs observed were a performative display to instill confidence in the Buddhist teachings and engender conviction and dedication in their practice. It was understood that he dutifully upheld the vows (*dam tshig*) he had received from his teachers—literally, *tuk dam* or one’s deepest heart commitments (*dam bca*’) to maintain a given practice and view.^29^ In doing so, this also represented a diligence in persevering to serve others since only with an enlightened mind could he take rebirth in a form that could be of greatest benefit to others. The date of his passing was also important because it was the anniversary of the Lhasa uprising the same year that he fled Tibet with the Dalai Lama into exile.

The Tukdam Study team was contacted. Yet due to lockdown conditions, the team could only coordinate body assessments after the 7th day post-clinical death and had no access to the study psychophysiological equipment, such as electroencephalograph, electrocardiograph, oximeter, capnograph, and skin temperature sensors. The team proceeded by instructing the attendant on the protocol using only a locally procured infrared thermometer and smartphone-procured images. The attendant was able to systematically procure daily images and video recording across each region of the body and sides with close-ups on face, limbs, chest, abdomen, hands, and feet.

Upon receiving the initial photographs even a full week into the postmortem phase, the team was struck by how fresh his body appeared. His skin was supple and warm in hue; limbs flexible and mobile; face pleasant, with eased expression as if he had just lied down for a nap. No sign of discoloration on any region of the body was visible marking a blatant absence of blood pooling on dependent surfaces that is characteristic even within the first hours after death and normally becomes fixed within the first day.^30^ The attendant had documented the room temperature maintaining a consistent range of approximately 70–74 ºF, and body temperature hovering at 72 ºF.

Image after image recorded this persistent state. Temperature, humidity, pressure readings of the room were taken and temperature readings on each region of his body logged the trend. The attendant reported that during daily body temperature assessments, he detected a higher temperature through gentle palpation with the back of his hand at the heart region compared to other regions of the chest and the lower abdomen. However, the infrared thermometer readings could not confirm this observation. Such tactile observations have been frequently reported in cases during the history of the Tukdam Study. In previous cases, infrared cameras had been employed to assess temperature differentials across the body, but no difference had been observed between the body and the ambient temperature in such cases.

Overall, the body temperature paralleled that of the room with minor fluctuations. Toward the 33rd day, the body demonstrated signs of warming to 77 ºF—then a paling of the skin, shifting coloration to a blue hue and then full presentation of cadaveric purple by the 36th day and fully present on the 37th day. Putrefactive blisters on limbs, localized cuticle detachment on fingers and mummification setting in at hands and feet also began in these last days. However, even on the 37th day, there was still no sign of bloating and the upper chest and abdomen still looked fresh. The upper torso exhibited the least visible change—significant for *tukdam* ontology where the heart center is understood as base for remnant mind activity and physical energetic support of body matter. Though no warmth could be detected, the resistance to decomposition in this region was considered an important indication that the subtlest level of mind still retained its subtle “wind mount” like a rider on a horse inside the heart’s indestructible essence.

Starting around the third week post-clinical death, the team observed the body start drying—slight indications at the tips of the fingers and around the cuticles at first, then gradually progressing up the hands, legs, and other peripheral body regions, respectively. However, none of these regions exhibited skin slippage and the skin across the central body retained elasticity and pliancy. The team was informed that the XIV Dalai Lama had begun reciting the Root Tantra of Guhyasamāja, a method for eliciting an individual to release *tukdam* in the Geluk School*.* A putrid odor was detected on the 37th day alongside diminished vibrancy in skin hue at the chest. The state was declared ceased and the body was prepared for cremation the following morning.

Despite the remarkable state exhibited across the previous weeks, upon cremation, Tibetan monastic and physician collaborators were surprised not to find characteristic relics in the ashes or observe distinct signs of clearing weather. However, the signs that occurred during this particular *tukdam* state were still considered exemplary.

It is the experience that this monk had with the practice of Guhyasamāja that was recognized as most noteworthy by the monastic and physician field members. Known as the King of Tantras, Guhyasamāja has been posited by XIV Dalai Lama as connected with extended *tukdam* states due to a practice focus on single-pointed concentration developed from techniques known as calm-abiding, or *śamatha*, and the complex detailed visualization of the specific maṇḍala in its final elaborate dissolution phase of the completion stage.

The generation stage purifies the process of birth and all one’s previous lifetimes’ births through a visualization of dissolving one’s mundane identity into emptiness and rearising through the self-visualization of the deity. However, the completion stage, most relevant to the context of *tukdam*, is understood to purify death since the practitioner engages the subtle anatomy of the pure form of the deity and dissolves that form into emptiness, allowing for recognition of the nature of mind as clear and luminous. It is this state that is recapitulated at the moment of death and in which a practitioner is understood to reside during *tukdam*.^31^

Though experience in completion stage practice is rarely admitted or openly discussed, the Gyutö monastic colleagues on the Tukdam Study team were able to learn that this senior monk received some of the most poignant completion stage instructions seen as particularly facilitative for entering *tukdam.* He received instructions on *tummo*, or inner fire practice, and vajra recitation, or a visualized syllabic mantra recitation between two facing deities, as in the Vajrabhairava and Guhyasamāja systems, respectively.^32^

Though case access was restricted and available investigative tools minimal, visual markers informed both the Euroamerican and Tibetan medical systems’ assessments of this state as exhibiting signs significant of a phenomenon distinct from the normal processes that unfold for a body when heart and lung function cease and activity in the brain dissipates.

## Biomarkers and Perceptual Cues in Biocultural Ontologies of Death

Although this case assessment began outside the temporal window in which rigor mortis is predicted to both develop and dissipate—similar to more than fifty other cases assessed by the study team thus far,^33^ the predictable postmortem chronological changes of pallor and livor mortis were suspended. This has been the longest case exhibiting attenuation of such signs, but has been a common observation documented across other *tukdam* cases in the study to date. Thus, the interest for the collaboration is in establishing a set of biomarkers recognized by the forensic field alongside perceptual cues accepted by the Tibetan tradition to signal the prominent chronology observed in these cases that appears distinctly outside the spectrum of normal postmortem chronologies accepted currently in forensic science (DiMaio & Molina, 2021, 20ff).

In the Tibetan Buddhist and medical context, an individual in the dying process is expected to first present with a loss of physical integrity and function of the organs and tissues. It will be followed by a desiccation of the body orifices, loss of body heat and then cessation of breath. Phenomenologically, the tradition describes the individual will experience a degeneration of the faculty of sight and quality of visual appearances, followed by deterioration of hearing, smell, taste, and touch. These progressions are understood to occur at coarse levels followed by subtle (Yutok Yönten Gönpo [12th cent. CE], 2006, pp. 35–41). For all individuals, specific activities in the subtle anatomy bring the subtlest level of mind into the heart region at which point an internal “breath” or wind is considered still present. Though this internal breath tends to remain only briefly for most healthy individuals, culturally, everybody is left for just over 3 days to allow the consciousness to transition into the intermediate state. The internal breath can remain substantially longer for advanced practitioners recognizing the nature of mind in the state of *tukdam*.

The clearest written historical accounts of the external signs of *tukdam* derive from Longchenpa and Tsongkhapa’s hagiographic biographies mentioned above, as well as Karma Chakmé’s *Mountain Dharma* retreat manual, and Jamyang Khyentse Wangpo’s arrangement of *Profound Instructions for Liberation Upon Hearing* (*Zab don thos grol*), a revealed treasure text of the Tukdrup Barché Künsel cycle of Chokgyur Lingpa.

In Longchenpa’s biography, the signs he exhibited, though not labeled *tukdam*, resemble what has been called *tukdam* since: a full lotus posture for 25 days; an odor “more fragrant than sandalwood or camphor”; meteorological and environmental signs, such as clear sky, rainbow light, and nearby flowers blooming; stronger concentrative stabilization and clarity of those meditating near, and so forth (Nyoshul Khen, 2005, p. 131).

In Tsongkhapa’s biography, the signs recognized during his tukdam were also full lotus position, radiant complexion that strengthened after death and youthful appearance. His skin is also described as firm, smooth, fresh and exuding a golden glow (Khedrup Je, 2021, p. 87).

Karma Chakmé (1613–1678), an accomplished scholar-yogin who wrote one of the most highly regarded meditation retreat manuals called *Mountain Dharma* (*Ri chos mtshams kyi zhal gdams*), warns us that an individual who enters *tukdam* may have more subtle signs than one might think (Khenpo Karthar, 2011b, p. 309). He says that many of the signs depend on the physical condition of the individual before they died. An upright posture of the individual, for example, he notes, could be an indication that attendants support the body well even if the person is not in *tukdam*. Likewise, if attendants are not good at supporting the body, the body could fall over even if the person is in the state (Khenpo Karthar, 2011b, p. 310). Reporting personally witnessing both contexts, he warns that upright posture is not a reliable indicator of *tukdam*.^34^

He also says that dietary intake, fasting, and an emaciated state will also affect physical expressions. Though fluid coming out of mouth, nose, or other orifices is thought to be a sign of the release of *tukdam*, it could merely be last intake excreted. Likewise, lack of fluid could result from a poor physical condition. Nevertheless, he says, for most conditions, clear, whitish, and/or reddish fluids emit from nose and mouth when *tukdam* releases; and for greatly realized, may contain *ringsel (ring bsrel)*, or spherical pearl-like relics (Khenpo Karthar, 2011b, p. 310). He describes in some cases when *tukdam* concludes, serous fluid emerges from the top of the head (Brahma’s aperture) and some emit urine or other fluids from the urethra.

Regardless of the cause of death or physical condition of the individual, he describes that the most certain signs of *tukdam* are a composed upright sitting posture held by the body itself because the individual has recognized and is resting in the ground clear light. Beyond this sign, he says the principle external sign is not of the body itself, but meteorological in content—the sky will be clear and cloudless, and in autumn, no frost. He describes that the most reliable indicator, and principal “internal” sign, is a healthy-looking complexion, “same as that of a living person” that remains through the period of *tukdam* (2011b, p. 311). For those who have been ill and lacked a healthy appearance immediately before death, they will often gain a more vital complexion during *tukdam*. In the classic delineation of external, internal and secret signs, that is roughly, those accessible to an external observer, those phenomenologically experienced, and those that arise as effects from accomplishment, respectively, he says that the principle secret sign will emerge when the individual is cremated as *ringsel*, spherical relics, syllabic seed forms, or other auspicious forms in the ashes.^35^

He adds several additional signs: eyes remaining half-closed, mouth smiling, nostrils not caving in, ears not closing or flattening out, and forehead veins still prominent. He describes the skin remaining elastic and pliable, springing back to original form after a pinch. He says that in summer, the body will not attract insects because it has not initiated decomposition; in winter, it will not freeze because it retains warmth. As an intersubjective sign, he notes, those who meditate in the individual’s presence will have clear meditation experience.

Karma Chakmé warns that upon entering *tukdam*, there should be no disturbance of the individual for the first week. He likens this to a small fire just lit that can easily extinguish. That is, the strength of the meditative state of clear light may not be strong enough to withstand external conditions. However, after a week, the fire will gain strength such that the clear light state cannot be disturbed even by major disturbances. The end of *tukdam* is indicated by loss of radiant complexion, sunkenness to the eyes, closing of the nostrils, and insects drawn to the body in summer and the body freezing in winter.

He warns that some cases that look like *tukdam* could be deceptive if the individual is not a realized practitioner and yet due to possession by a spirit, the body retains a *tukdam*-like upright posture and strong complexion.^36^

In the next text that most explicitly details the signs of *tukdam* recognizable by an external observer, the *Profound Instructions,* like Rigdzin Nyima Drakpa’s arrangement of *Great Liberation Upon Hearing* described above, is meant not only to be studied by practitioners, but actually recited to those individuals amidst the dying process itself to guide the transition into intermediate states where the consciousness, having released the support of the body for that lifetime, journeys to the next rebirth. Recognizing if the individual is in *tukdam*, then, allows for one to determine whether any support is necessary to be read from the liturgy, and how to recognize the state has concluded to determine when a cremation would be appropriate.

In this text, the dissolution of *tukdam* is described as concurrent with signs that the inner breath has ceased, specifically the sign of fading of the body’s warmth (*drod*) and the sign of diminishing radiance (*mdangs*), similar to the description given by Karma Chakmé. This loss of heat, though not specified as occurring in a particular region, given the context of the passage, refers to the site of the inner breath, which is at the heart. The accompanying radiance, likewise, is understood to stem from the heart center but permeate systemically as well. If the *tukdam* goes too long, the tradition also has a separate textual recitation for releasing the state.^37^

Despite the various signs described by Karma Chakmé and Jamyang Khyentse Wangpo above and insinuated in both Longchenpa’s and Je Tsongkhapa’s hagiographic biographies, the release of *tukdam* in its definitive form is recognized in practice as a slumping of the body (for those in upright unsupported meditation posture), the appearance of a putrid odor, and initial signs of decomposition (Dalai Lama XIV, 2021). All such signs are detected as perceptual cues that the underlying state of meditation has ceased.

## Bridging Epistemologies, Mapping Distinct Ontologies

What epistemological bridges might a collaborative inquiry into *tukdam* create? What are the signs recognized by one intellectual tradition with an epistemology attached to external instrumentation that allows them to say a state of being is significant compared to the perceptual cues of another tradition that even relies upon a set of more ephemeral signs such as dreams, contemplative insight, and expanded awareness to indicate supramundane states? In the case above assessed amidst the unique conditions of COVID-19, both teams had to settle on a similar means of investigation—visual metrics for tracking postmortem physiological changes. However, in other contexts, documentation of visual details would rarely be sufficient evidence to substantiate a claim of an unusual postmortem state. Only invasive measures to procure fluid and tissue samples for analysis or deployment of sorbent tubes to capture volatile organic compound emissions would make a viable case; and EEG, EKG, and oximetry is only relevant during and immediately after clinical death. However, without access to such equipment and with limitations on cultural acceptability of methods used, the two intellectual traditions agreed upon an approach that, by default, privileged the epistemology of the Tibetan tradition by relying on perceptual cues.

The Tukdam Study has been like a psychophysiological expedition where two field teams traverse identical landscapes with one team in a thick-walled vehicle with the highest technology to measure altitude, slope, inclination, moisture, and so forth to navigate. Whereas the other team is out in the elements of the environment fully exposed but able to directly see every microscopic change in vegetation, geography, weather, and incident. The latter team being autochthonous to the landscape—the cultural phenomena observed—tries to explain to the team inside the vehicle what they are observing through unmediated means. Both have access to an identical map on which they are referencing similar landmarks and signs, but each are also adding to their individual map the signs that make sense to them from their respective instrumentation—one, technological eyes, ears, nose, and hands and the other, perceptual. The two traditions can speak to one another about their landscape, but they feel as if these landscapes are not identical because both exploratory means or investigative vehicles with which they journey across the landscape are distinct and the maps each have augmented also now quite different.

This community-based participatory research entreats epistemological participation from both sides—the neuroscientists in the contemplative field of exploration and the Tibetan monastics and physicians in the neuroscientific field of electrical impulses as brain, heart, breath, and life. Each participates in co-developing constructs that the team as a whole is analyzing and each helps implement specific ways in which the measures elucidate those constructs—the proverbial landmarks they place on their individual maps. It is a true cross-cultural collaboration, guided by some of the greatest minds in each community, yet also like dancers from two different traditions, say, tango and salsa, cuing off different leads of respective torso and hips, there seems to be an asynchrony in the attempts of a coordinated dance.

Both Tibetan medical and Euroamerican biomedical systems rely on indicators from each tradition’s respective ontological paradigms and epistemic diagnostic instruments to guide recognition of phenomena as mundane or supramundane. On one side are biomarkers as objective measures of observable physical changes. On the other side are perceptual cues with content, at times, synergistic to the external observable markers of the Euroamerican system, and at other times, phenomenological, even intersubjective and ecological, in perceived effects. Both lenses seek to track specific processes and progressions through a set of signs that can be tracked both during the dying process but also as confirmation of the final transition to death—or transcendence, from the Tibetan ontological view. The postmortem chronology most widely accepted by Euroamerican biomedical and forensic communities relies on biomarkers that index an assumed relatively homogenous transition at the end of life from which decompositional factors are unconditioned by the qualities of the entity that previously enlivened the material form. Tibetan medical physicians and Buddhist adepts, on the other hand, rely on perceptual and inferential markers that do not necessitate a disentanglement between body, mind and consciousness. Quite the contrary, with greater levels of subtle activity, they assume their coincidence and mutual influence (Ozawa-de Silva and Ozawa-de Silva, 2011). Through this perspective, the subtle remnant consciousness integrally induces a plasticity of chronomorphological effects on the physical body after clinical death is expected, let alone a variety of phenomenological experiences of the transitioning consciousness.

Over the last decade in contemporary research in collaborations between the Tibetan and Euroamerican traditions, Euroamerican epistemologies and modes of inquiry have dominated. This has resulted in relegating traditional modes of inquiry in the Tibetan system, such as direct perceptual evidence and inferential interpretations, to largely theoretical bystanders. Yet this novel development in building epistemic bridges vis-à-vis the Tukdam Study has allowed the various collaborator groups to explore the ontological realities at play for each system and the variations on the biocultural nexus of life and death that each perceives. It allows them to explore the breadth and diversity of perceptual signs such as radiance, heat, and posture alongside biomarkers such as emitted VOCs, microbiotic changes in orifices and epithelial surfaces, and degrading DNA, RNA and proteins—as well as EEG-measured brain activity and oximetry, if a close temporal window permits. It also posits the possibility for discourse by scaffolding those epistemic bridges, even for supramundane signs such as post-cremation relics.

## The Short Yet Miraculous Bounty Case

In November 2021, another *tukdam* case took place. This time it was a young Rinpoche age 37 years old for whom his attendants and family could not attest to his practice of highest yoga tantra cycles. They spoke of his kind heart, his humility, his reverence, and devotion to his teachers. Still in the context of COVID-19 restrictions and equipment use limited to images and temperature readings, when the Tukdam Study team first received images of his body, there were clear indications it had already advanced to cadaveric blue discoloration from head to toe. It was declared by expert monastic and Tibetan medical colleagues not to be a *tukdam* case. Yet, unlike the previous case for which little sign emerged after his cremation, this case produced a bounty of pearl-like *ringsel* and other *kü-düng* (*sku gdung*) relics, representative preserved forms of the decedent’s pure body coming out of the ashes from the fire, as described above as a principal sign of achievement by Karma Chakmé. One of the most striking emanations was his skull inscribed with signs of mantra and deity, representative of a highly detailed and nuanced maṇḍala from the highest yoga tantra—his ontological form manifested into a kaleidoscope of enlightened emanations, like a dance of the deities of Guhyasamāja. This display was one of the most remarkable for our Tibetan Buddhist and medical colleagues. Such post-cremation forms were the first of their kind witnessed by our Russian collaborators. Astounded, they were eager to apply biochemical analyses to these physical emanations, yet the attendants reverently carried them off for enshrinement.

## Conclusion

In this context of “life suspended in death,” teams from two distinct intellectual traditions, several members of which have trained in both knowledge systems, apply a practice of building epistemic bridges to cultivate biocultural lenses—both Euroamerican and Tibetan—through biomarkers and perceptual cues attendant to their respective ontologies that frame the investigative object simultaneously as dying person, a moment of awakening, and a transitional state from life to death to luminosity. These two cases highlight particular distinctions of the interface between a given intellectual tradition’s epistemology—the instrumentation with which it seeks to access knowledge, and its ontology—the way in which it perceives the living forms it engages, and the attendant properties and relations between them. The case of *tukdam* demonstrates how this collaborative research approaches the development of constructs and measures that link biomarkers in the Euroamerican tradition with perceptual cues in the Tibetan Buddhist and medical tradition to traverse a common landscape, though with distinct vehicles, that inform the maps each create of those realities.

## Footnotes

1 Of note, Buddhism and science collaborations and cross-training of intellectual traditions are not universally accepted across Buddhist institutions due to the significant time monastics must dedicate to their science studies in such programs. Likewise, only a limited group of scientists are interested in engaging in such dialogues, and the significant investment scientists must make in Buddhist philosophical and practice studies to productively engage in partnered research and discourse also provides a significant barrier. There is also resistance within the Tibetan Buddhist community to the idea that science must ‘prove’ Buddhist insights on well-being, and resistance within the scientific community that Buddhist thought has novel perspectives to provide science. Nevertheless, fruitful outcomes on both sides have been described (see, for instance, Dalai Lama et al., 1999; Dunne and Goleman, 2018; Dalai Lama XIV, 2018, 2020b; Worthman et al., 2021; Gray et al., 2022).

2 E.g., for Geluk and other ‘new school’ views, see Je Tsongkhapa ([14th cent. CE], 2013) detailing Guhyasamāja-based descriptions; for Nyingma and other related ‘old school’ views, see Padmasambhava ([8th cent. CE, 14th cent. CE extant], 2007) detailing Guhyagarba-based descriptions.

3 For ethnographic accounts, see Zivkovic (2010, 2013), Coleman (2017, this issue), Namdul (2019, 2021, this issue), and Lott (this issue).

4 Explicit references to the term *tukdam* as a meditative state accomplished in the moments of death are found in numerous sources with internal subjective signs described. However, few sources detail the specific external signs accessible for a third person observer to evaluate.

5 *Bar do thos grol chen mo,* hereafter referred to as *Great Liberation Upon Hearing.*

6 A treasure-revealer is considered a realized master whose agency is critical for the re-emergence of Revealed Treasure texts. The treasure tradition (*gter lungs*) plays a central role in the Nyingma School of Tibetan Buddhism in which the texts are considered to be originated by the Indian master Padmasambhava who established Buddhism in Tibet.

7 *Zab chos zhi khro dgongs pa rang grol,* hereafter referred to as *The Peaceful and Wrathful Deities.*

8 Three chapters of the edition were translated by Kazi Dawa Samdup and edited by Walter Y. Evans-Wentz in 1927 to produce the English text that populated widely into circulation. All twelve chapters have been subsequently translated (see, for instance, Padmasambhava [8th cent. CE, 14th cent. CE extant], 2007). See Lopez (2011) for the historical context for Evans-Wentz’s decontextualization of the text as a universal “art of dying.”

9 These purportedly phenomenological accounts affirming the signs depicted are also complemented by a rich autobiographical literature of those who “came back from death,” or *délok* (*’das log*), and provided descriptions of their experience. For historical and socio-religious accounts of such narratives, see Epstein (1982, 1990), Pommaret (1989, 1992, 1997, 2012), Cuevas (2008), and Galli (2021). Of note, though *tukdam* cases are predominantly male due to the gendered context in which individuals in Tibetan contexts gain access to advanced practice, *délok* accounts are largely female (Prude, 2016).

10 As written by Chödrak Zangpo (14th cent CE, TBRC W2CZ7505), and later by the Fifth Lhalün Tuksé Kunzang Gyurmé Chokdrup in 1725 (Sönam Chödrup, 1994); also notes inscribed by a disciple after Longchen Rabjam’s last testament in *Stainless Light* (*Zhal chems dri ma med pa'i 'od*), folio 132b/5, Khadro Nyingthig, Vol, Hum (Adzom edition), noted in the first in the series of the translation of the *Seven Treasures* (*Mdzod bdun*) (Barron, 1998, xv).

11 Known as the *Trilogy of Dispelling Darkness* (*Mun sel skor gsum*).

12 Because Longchen Rabjam Drimé Özer's final transcendence was done in a large gathering and the great transference into a rainbow body (*’ja’ lus ‘pho bac hen po*) is meant to occur in a more private way [Yangthang Tulku in *The Nyingthig Yabshi Empowerments* as described by Stewart (2014)], his transition exhibited signs characteristic of *tukdam* instead.

13 For further descriptions of these two stages, see Tsongkhapa (2013).

14 Thirty-two deities in the Ārya tradition where the central deity is blue-black Akṣobhyavajra, a form of Akṣobhya, one of the five tathāgathas; nineteen deities in the Jñānapada tradition, where the central deity is yellow Mañjuvajra, a form of Maṇjuśri with three faces that represent the three main channels of the subtle body and the three stages of purification of the mind or the illusory body, light, and their union (Kilty 2013, p. 9).

15 For a contemporary Tibetan Buddhist modernist understanding of the brain and death process comparing Tibetan Buddhist and Euroamerican views on the subject of rebirth, see Khenpo Tsultrim Lodro’s (2003) *The Mirror That Illuminates Existence: An Analysis of Past and Future Lives* (2003), as analyzed in Sheehy (2021).

16 The equivalent in the context of diabetes mellitus would be HbA1c as a marker of cumulative effects on longer time courses, e.g., 3-month periods of glucose regulation.

17 Technically, this occurs through a process of cognitive exclusion called *apoha*, in which all particulars of perceptual input are cognitively excluded except those relevant to the underlying goal in the process of conceptual cognition (Dunne 2011; Eltschinger et al., 2018, p. 3). Practical activity, as Prueitt describes, proceeds on the basis of these phenomenal forms as a means of trustworthy awareness (*pramāṇa*) that, once conceptualized through the *apoha* process, produce a determination that leads to the accomplishment of the perceiver’s goal (2017, pp. 23–24).

18 For the species relevant to this region of India, see Bharti and Singh (2003), for those relevant to south India, see Byrd (2009, p. 375).

19 Mapped into a Glaister equation or nomogram, assuming a normal temperature at death, predictable pattern of cooling and a standard subsequent temperature increase (Henßge & Madea, 2004).

20 Based on the amount and type of food present in the gut at death to predict time since death (DiMaio & Molina, 2021, p. 31).

21 DNA, RNA and proteins tend to degrade at predictable rates by proteases, nucleases and bacteria (Choi et al., 2019; Rhein et al., 2015; Sampaio-Silva et al., 2013). MiRNA profiling can also identify fluid type. As small non-coding RNAs with approximately 22 nucleotides of length, miRNAs have signature markers for the major body fluids, such as saliva, serum cerebrospinal fluid, peritoneal fluid, venous blood, plasma, semen and vaginal secretions (Silva et al., 2015, p. 3).

22 The thanatomicrobiome delineates expected microbial community changes in the buccal mucosa and other body fluids and tissues providing a microbial clock indicate time elapsed since death (Javan et al., 2016, 2019).

23 VOC composition and rate for different postmortem stages have provided an increasingly utilized marker for time since death (Vass 2019; Verheggen et al., 2017).

24 For example, concentration of vitreous potassium is controlled by decomposition so slowed rates will result in low potassium levels providing an inaccurate method for determination of time of death (DiMaio & Molina, 2021, p. 29). Likewise, despite positive correlations between time of death and amount of degradation of DNA, RNA, and protein in body fluids, the predictive function is too variable for practical use, let alone equipment access demands of such techniques. Digestion and fluid excretion also exhibit great variability. The latter three measures—molecular degradation in fluids, thanatomicrobiome changes, and VOC emission characterization would be helpful measures for the project under conditions where fluids could be procured and sorbent tubes administered, and analyses compared to baseline data for each marker.

25 Although an opto-electronic device measuring the color shift across skin tone has been used by forensic pathologists for assessing postmortem morphological changes, it is only accurate for light or white skin tones (Schäfer, 2000). Such a device is not suitable for the range of skin tones across *tukdam* cases.

26 A publication from the collaboration presenting case study results using this forensic method is forthcoming (Tidwell et al., forthcoming).

27 Some early Buddhist textual examples for the study of iconometric proportions include the *Mañjuśri Mūlakalpa*, *Samvarodaya*, *Kṛṣṇayamāri*, and *Kālacakra* tantras (Jackson & Jackson, 1984).

28 Practitioners from different Buddhist schools are described to enter *tukdam* in different ways. For example, Ganor Dzokchen Rinpoche describes that the preliminary practices a practitioner undertakes will distinguish the length of time that a practitioner will remain in *tukdam*, based on the vows related to that practice (Ganor Rinpoche, 2013). Dzitok Rinpoche describes that it also refers to the heart-vow (lit., *thugs dam*) to reside in the natural state of the mind, recognizing the *dharmakaya*, or truth body, as the fundamental state of reality or existence (Dzitok Rinpoche, 2013).

29 *Tukdam* is also a term for one’s chosen deity (*yi dam*) or routine practice of meditative state that can accomplish the central aims of tantric or high meditative practice.

30 “Fixed” refers to the blood pooling reaching its maximum coloration.

31 Of note, this description of the generation and completion stage practices applies to all highest yoga tantra classes, not just the Guhyasamāja.

32 Though the Kālacakra system also has importance to the Geluk School, from the point of view of the Guhyasamāja system, the Kālacakra system focuses on the dematerialization of the form aggregate rather than the manifestation of an illusory body as achieved in the intermediate state. Thus, it has been hypothesized that the type of practice the Kālacakra system facilitates requires realization in the context of a coarse body (Cozort, 2005, p. 130), and therefore its practitioners would not be predicted to exhibit manifestations of *tukdam* in the way predicted by accomplished practitioners of the Guhyasamāja system. The pinnacle of realization for the Kālacakra system is a body of light (Lamrimpa, 1999, p. 175) similar to the rainbow body (’*ja*’ *lus*), or great transference into the rainbow body (*’ja’ lus ’pho ba chen po*), in Dzogchen systems. In the case of the rainbow body, the five gross elements that form the physical body dissolve into their essence—five-colored light appearing as a rainbow mass of light, leaving only hair and nails behind. Thus, no corpse would remain unlike the case of *tukdam*. As one of our anonymous reviewers skillfully pointed out, an important area of investigation that is beyond the scope of the current paper is investigating the ways in which the illusory body practice of the Guhyasamāja system could be distinct such that it is associated with *tukdam*, and how other tantric systems applying the illusory body practice from the Six Doctrines of Nāropa or Six Doctrines of Niguma, might not have the same association with *tukdam*. It has been posited that the extensive maṇḍala of Guhyasamāja and the stable *śamatha* it cultivates might play an important role in the extended *tukdam* states associated with this practice.

33 Results from these cases are currently under review and in development for publication; with plans for future publications on assessments from the other physiological measures.

34 Notably, recent authoritative accounts have, in fact, used the upright posture to distinguish ‘definitive tukdam’ (*thugs dam mtshan nyid pa*) from ‘common tukdam’ (*thugs dam phal ba*) (Samdhong Rinpoche, 2017; Ngawang Jinpa, 2020, p. 513).

35 Karma Chakmé describes types of *ringsel* in Mountain Dharma (2011b, pp. 315–319).

36 Tibetan lore narrates stories of such cases where the individual actually transforms into a zombie, and begins to speak, whistle, or whisper. Karma Chakmé notes such clear descriptions in the Six Doctrines of Nāropa, where the consciousness of another temporarily makes use of the body since the person’s consciousness has already departed—a case of spirit possession.

37 *Thugs dams grol ma pa*, personal communication Phakchok Rinpoche, April 2023.
