# Correction: Life in Suspension with Death: Biocultural Ontologies, Perceptual Cues, and Biomarkers for the Tibetan Tukdam Postmortem Meditative State

**DOI:** 10.1007/s11013-025-09935-2

**Published:** 2025-07-24

**Authors:** Tawni L. Tidwell

**Affiliations:** Center for Healthy Minds, University of Wisconsin-Madison, 625 W. Washington Ave., Madison, WI 53703 USA

**Correction to: Culture, Medicine, and Psychiatry** 10.1007/s11013-023-09844-2

In the original publication of the article, abstract section has been unfortunately published with errors. The corrections are listed below:

1. in line 2 of the abstract section, the words “tukdam (thugs dam)” should be “*tukdam* (Tib., *thugs dam*)”.
2. in line 7 of the abstract section, the word “traditionsenact” should be “traditions enact”.
3. in line 14 of the abstract section, the word “epistemologicalinquiry” should be “epistemological inquiry”

The original article has been corrected.

